# Creating a small baby program: a single center’s experience

**DOI:** 10.1038/s41372-021-01247-8

**Published:** 2022-01-01

**Authors:** Anamika I. Banerji, Andrew Hopper, Munaf Kadri, Benjamin Harding, Raylene Phillips

**Affiliations:** Division of Neonatology, Loma Linda University Children’s Hospital, 11175 Campus Street, Coleman Pavilion Ste 11121, Loma Linda, CA 92354 USA

**Keywords:** Outcomes research, Ethics

## Abstract

Creation of a small baby program requires special resources and multidisciplinary engagement. Such a program has the potential to improve patient care, parent and staff satisfaction, collaboration and communication. We have described benefits, challenges, and practical approaches to creating and maintaining a small baby program that could be a model for the development of special programs for other sub-populations within in the NICU.

## Introduction

### Why have a small baby program?

The percentage of preterm infants born at extremely low birth weight (ELBW) defined as <1000 grams has not changed significantly over the last several decades; however, the total number of ELBW births has increased [1]. Evidence of increased risk for long term challenges after discharge in this population has been well documented for many years [2].

Caring for this population offers many challenges, including ethical dilemmas related to peri-viability, when to offer perinatal monitoring and resuscitation, reaching consensus in developing and following guidelines, and resolving conflict when parental wishes do not align with the medical team’s plan of care. Medical care for ELBW infants requires understanding complex pathophysiology in fragile infants with suddenly and frequently changing requirements. Since neonatology training includes care for ELBW babies, the question about the need for dedicated small baby programs has been raised. Is there evidence to support a focused approach to care of this population?

Collaborative interdisciplinary models of standardized care have been shown to improve patient outcomes, as well as parent and staff satisfaction [3]. Nationwide Children’s Hospital was among the first institutions in the United States to demonstrate that standardized care was one of the key components to improving morbidity and mortality of extremely low birth weight (ELBW) infants [4]. Regional care approaches have demonstrated that facilities and healthcare practitioners who see more patients with a particular pathology have better outcomes than those who rarely treat this population [5–7].

The most compelling rationale for having a small baby program is that efforts to ensure the successful survival and discharge of ELBW infants without major disabilities may be best framed within a dedicated program focusing the collaborative efforts of many on a shared goal.

We hypothesized that developing a dedicated small baby program to provide standardized, comprehensive interdisciplinary care would improve safety through focused quality improvement and would ultimately improve morbidity and mortality for ELBW infants in our care.

While we are in the process of collecting data to support our hypothesis, in this paper, we describe our experience of creating a small baby program in our 84-bed, Level 4B NICU at Loma Linda University Children’s Hospital, an academic teaching hospital located in Southern California. We share our perspectives on the planning processes, challenges during implementation, solutions that emerged, lessons learned, and future directions for the purpose of supporting others with similar goals.

## Process of creating a small baby program

In designing and implementing a small baby program, we identified several areas of focus and approached our processes in three phases described below.

### Phase I: needs assessment and administrative support

The first phase in our process was to perform a needs assessment to determine (1) what components would be necessary to create a successful small baby program, (2) was there a dedicated space that would be suitable for a small baby program at our hospital, and (3) would hospital administration support a small baby program?

After performing a literature search and visiting several regional NICUs with successful small baby programs, we approached hospital and finance leadership with our assessment of the need to create a small baby program at our hospital. The higher acuity of this fragile population necessitated greater medical knowledge and expertise from bedside providers to manage complex and fluctuating pathophysiology. The need to provide additional education, training, supervision, and graded autonomy for medical trainees regarding ELBW pathophysiology, proficiency in life-saving procedures, and providing neuroprotective nursing and respiratory cares were our areas of focus. We identified a separate space near our NICU that, with modifications, could be used as a small baby unit. After the cost-benefit analysis was completed, our hospital administration made a commitment to support the program.

### Phase II: achieving interdisciplinary investment & implementing foundational principles

The second phase in our process included achieving an interdisciplinary shared model of care that included specific principles unique to this population. We identified a core group of disciplines directly involved in the care of ELBW babies and shared the concepts involved in a small baby program. Achieving interdisciplinary investment from this group was necessary for the success of our program, which could not be done without a collaborative effort. Creating an effective program required explaining the program’s rationale, modifying workflows, protocols, and practices for all groups working in concert.

To reinforce the accountability of leadership in the necessary disciplines, we established an interdisciplinary general steering committee that met twice monthly. This group served as planners and disseminated information within each discipline. A small baby medical subcommittee was formed to standardize medical protocols. A small baby nursing subcommittee was created to review small baby care practices from across the country and update and teach appropriate nursing practices.

### Defining our population

How small is too small? [8, 9]. Invested stakeholders reviewed the available literature. Guided by a national consensus statement [10], we chose to initiate steroids and expectant management at 22 weeks 5 days gestation and full NICU resuscitation beginning at 23 weeks 0 days gestation. While making provision for exceptions, implementing standard guidelines helped guide discussions between providers and families.

We developed specific admission criteria using both gestational age (<30 weeks) and weight (<1000 g) for inborn and outborn infants less than seven days old and cohorted these infants in the separate space dedicated for small babies. Cohorting was challenging because it prohibited pairing with less acute babies, requiring more 1:1 staffing ratios. Although initially increasing nursing staff cost, this change in staffing resulted in fewer complications (IV infiltrates, skin tears, delays in feedings or medications, etc.) by making it possible for nurses to provide more focused attention to their more vulnerable patients.

### Creating dedicated space for small babies

A space for our small baby unit separated from the general NICU population created a physical and psychological environment more suited to the developmental needs of ELBW infants. Based on successful design features of other units and previously published guidelines [11, 12], we focused on reducing ambient noise and light, optimizing nursing workflow, and providing adequate bedspace and storage needs.

### Neuroprotection family-centered developmental care

Because developmental care practices improve outcomes for preterm babies [13–16], we used the Neonatal Integrative Developmental Care model to implement principles and practices of the Seven Core Measure of Family-Centered Developmental Care [17]. A key element in this model is recognizing the essential role of parents [18–20] in the lives of preterm babies to support physiologic stability, healing, growth, and optimal cognitive and emotional development.

### Interdisciplinary small baby education

Our interdisciplinary program required specialized education to learn the principles important to the success of caring for this population. Educational curricula included four main components.

1. Pre-reading Articles and Chapters: Assigned reading focused on pathophysiology of ELBW infants and evidence-based interventions and care practices for this population.
2. Pre-recorded Didactic Modules: Topics of particular importance for our unit were described in more detail.
3. Interactive Simulation/Stations: Five stations included: (a) neuroprotection skills, (b) delivery room practices, (c) respiratory practices, (d) nursing practices, and (e) case reviews. NICU physical therapists demonstrated how to apply neuroprotective care principles, including two-person cares. A multidisciplinary delivery room simulation training focused on a hands-on resuscitation of ELBW infants using a high-fidelity electronic simulation doll [Premature Anne, Laerdal] to practice the tasks and roles in ELBW resuscitations. NICU respiratory therapists and nursing leadership discussed and demonstrated their role-specific protocols and care techniques. A final station allowed for case-based review of common diagnoses and challenges and a new approach to interdisciplinary rounding [21].

### Implementing standardized guidelines

Standardized guidelines were created for major areas of small baby care.

#### Delivery room care

This portion of the education highlighted the importance of teamwork [22]. Experienced attending physicians and advanced-practice clinicians were expected to execute delivery room practices that were specific for ELBW infants. They performed a checklist-guided pre-brief with the team, partnered with OB to handle, position, and wrap the delivered ELBW infant in a neuroprotective, developmentally-appropriate manner and guide delayed cord clamping in the delivery room, then gently moved the infant to the NICU team who would oversee timed, recorded NRP-guided resuscitation efforts guided by role-specific multidisciplinary small baby checklists [23–25].

#### Routine infant cares

To protect infant sleep for healing, growth, and brain development, we enforced clustered care every four hours for routine cares and examinations. We implemented a care practice of two-person/four-handed support where one individual provides flexed/tucked infant containment for the baby (mimicking womb positioning), while the second person performs cares, procedures, or exams [13, 26–28]. Pauses are provided in response to infant stress to allow recovery. While it took pre-planning to time routine exams with cares, this approach resulted in a significant decrease in apnea, bradycardia, and desaturation events than previously observed.

#### Mechanical ventilation

Our approach to mechanical ventilation became more uniform with the small baby program. We chose volume-targeted ventilation as the primary strategy for newly intubated ELBW infants [29]. Bubble CPAP was continued as our primary non-invasive ventilation modality, using non-invasive intermittent ventilation (NIMV) as a bridge. Compliance with these practices is reviewed on daily bedside rounds. We implemented histogram cards, in which our nurses report percent of time each infant spends in target saturation range over the last 12–24 h. These data will be linked to retinopathy of prematurity rates.

#### Patent ductus arteriosus (PDA)

We reviewed the available literature and national practices and developed an algorithm to standardize our PDA management, which has been audited frequently and updated through several Plan-Do-Study-Act (PDSA) iteration cycles.

#### Feeding Protocols

We focused on using our existing standardized feeding protocol to advance and fortify feeds more uniformly and reinforced guidelines for central line discontinuation. We also addressed more optimal support of mother’s milk supply and use of donor milk when mother’s milk is not available.

#### Incubator humidity

A standardized approach to management of incubator humidity allowed us to reduce delivery of excessive fluids while preventing hypernatremia and, in some cases, acute kidney injury related to volume depletion.

### Focusing on interdisciplinary communication

Communication is essential to create a unit culture around small baby care and to support standardization of new protocols and practices.

#### Structured interdisciplinary bedside rounding (SIBR)

We modified our daily rounds to give nurses, respiratory therapists, and parents an active role in a process known as Structured Interdisciplinary Bedside Rounding (SIBR) [30]. To optimize bedside staff engagement [31], we created specialized templates for nurses and respiratory care practitioners as prompts for SIBR rounds. SIBR rounds improved efficiency (decreasing “surprise orders”), prompted intersystem discussions, and enforced guidelines. Since parents are included in SIBR rounds, this process improved parent and staff satisfaction and understanding of team goals.

#### Interdisciplinary communication challenges

With a large NICU staff, communication challenges have been significant. As protocols are evaluated and adjusted, communication about these changes is critically important for continued uniformity in care. It is equally important to provide a forum for and respond to staff concerns in a timely manner. We have used educational sessions, email, a shared computer drive, private social media forums, newsletters, flipcharts, and our division’s intranet to share conversations, concerns, and updated protocols.

### Phase III: data collection, QI, and research initiatives

Acquainting all staff with new practices has led to the inevitable questions: are they working? Do we have data supporting their continued practice? Are there better ways of achieving improved outcomes? As we evaluate our practices, we are engaging in a multitude of quality improvement (QI) and research projects. In addition to supporting evidence-based care, these projects provide opportunities for residents and fellows to fulfill educational requirements. Because this is an interdisciplinary endeavor, nursing and respiratory care participation are actively sought, thus supporting increased investment in the success of our small baby program by everyone involved.

## Discussion

What have we learned? Our center’s experience in opening a small baby program has been challenging and exciting. Our experience highlights central tenets to success, including hospital administrative and interdisciplinary support, resource investment, prioritizing ongoing education and communication, and reinforcement of standardized practice through consensus and commitment to this model.

The interdisciplinary aspect of the small baby program led to creativity in several aspects of patient care including innovative strategies for care of very small nares and less invasive surfactant delivery, as well as developing interdisciplinary checklists for catheter-based PDA closure. Cohorting was a key step that led to many creative improvements. Caring for the same population daily, highlighted reoccurring challenges often responding to the same solutions, which would have been less easily recognized in the traditional model of non-cohorted infants spread across different teams with frequent changes in providers.

In planning and evaluating our small baby program, an important concept has emerged. It seems apparent that while all NICUs may care for some small babies, not all NICUs caring for small babies should seek to establish a small baby program. Our experience has reinforced our perspective that small baby program requirements should include administrative support, a physically dedicated space within a larger general NICU, standardized protocols, physician, nursing and respiratory staff commitment to this model, specialized equipment and adequate financial resources to teach and maintain competencies, provide higher staffing ratios, invest in technologies such as near-infrared spectroscopy (NIRS) and amplitude integrated EEG (aEEG) used for management and research.

A critically important aspect of a successful small baby program is the dedicated education and support for small baby staff. Taking care of this high-acuity population is taxing and the potential for burnout is high. Ongoing communication, support, and sharing of goals and outcomes is vital. This is confirmed in the VON experience, showing that trust, collegiality, and transparent outcome-sharing bolstered better practices [32]. It was determined that camaraderie, morale, and commitment to problem-solving correlated significantly with mortality-morbidity reduction. These principles have inspired and motived us in the development of our small baby program with the same goal to reduce mortality-morbidity in this especially vulnerable population in our NICU.

## Conclusion

Our group has experienced great satisfaction and humility in creating a small baby program. We are daily inspired by our small patients and their families to implement and constantly re-evaluate best practices. The initial and on-going efforts have been significant. However, we have found that satisfaction in the process counters burnout and fatigue, providing its own reward with invaluable, if less measurable benefits, that may be critical rubrics of success in the future [33]. Our early work confirms the insights shared in studies of other high-performing NICUs [34] and endorses the concept, process, and required resources needed for a small baby program.

We are in the data-collection phase of our program, and moving forward, we hope to collaborate regionally and nationally with other successful small baby programs to standardize the definition of a small baby program, to share management strategies, and collaborate on relevant research and quality improvement matters. As more outcome data is made available, the success of small baby programs may establish with even more certainty, the need for specialized care in this vulnerable NICU sub-population and may serve as a model for improved care of other sub-populations.
